# Basolateral Amygdala Inactivation Impairs Learning-Induced Long-Term Potentiation in the Cerebellar Cortex

**DOI:** 10.1371/journal.pone.0016673

**Published:** 2011-01-31

**Authors:** Lan Zhu, Tiziana Sacco, Piergiorgio Strata, Benedetto Sacchetti

**Affiliations:** 1 Department of Neuroscience, University of Turin, Turin, Italy; 2 National Institute of Neuroscience-Italy, Turin, Italy; Pontifical Catholic University of Rio Grande, Brazil

## Abstract

Learning to fear dangerous situations requires the participation of basolateral amygdala (BLA). In the present study, we provide evidence that BLA is necessary for the synaptic strengthening occurring during memory formation in the cerebellum in rats. In the cerebellar vermis the parallel fibers (PF) to Purkinje cell (PC) synapse is potentiated one day following fear learning. Pretraining BLA inactivation impaired such a learning-induced long-term potentiation (LTP). Similarly, cerebellar LTP is affected when BLA is blocked shortly, but not 6 h, after training. The latter result shows that the effects of BLA inactivation on cerebellar plasticity, when present, are specifically related to memory processes and not due to an interference with sensory or motor functions. These data indicate that fear memory induces cerebellar LTP provided that a heterosynaptic input coming from BLA sets the proper local conditions. Therefore, in the cerebellum, learning-induced plasticity is a heterosynaptic phenomenon that requires inputs from other regions. Studies employing the electrically-induced LTP in order to clarify the cellular mechanisms of memory should therefore take into account the inputs arriving from other brain sites, considering them as integrative units. Based on previous and the present findings, we proposed that BLA enables learning-related plasticity to be formed in the cerebellum in order to respond appropriately to new stimuli or situations.

## Introduction

In fear conditioning, a neutral stimulus (conditioned stimulus; CS), usually a light or a tone, is presented in conjunction with an aversive event (unconditioned stimulus; US), typically footshock. After pairing, the CS acquires aversive properties and will, when presented alone, elicit a host of species-typical defense responses, including freezing, alterations in autonomic nervous system activity, neuroendocrine responses and potentiation of reflexes. It is now well established that different aspects of fear memory are distributed in multiple brain memory systems [1]–[4].

Cerebellar cortex participates to learned fear [5]–[6]. Lesions of the cerebellar vermis affect conditioned fear responses without altering baseline motor/autonomic responses in animals [7]–[8] and humans [9]. Reversible inactivation of the vermis during the consolidation period impairs subsequent retention of fear memory [10]. In humans, cerebellar areas around the vermis are activated during mental recall of emotional personal episodes [11], if a loved partner receives a pain stimulus [12], and during learning of the association between sensory stimuli and noxious events [13]–[14]. It has been proposed that cerebellum learns and retains fear memories in order to set the more appropriate responses to a new stimuli and/or situations [11].

In the cerebellar cortex, fear learning induces a synaptic strengthening at the parallel fibres (PF) to Purkinje cells (PC) synapses strictly related to associative processes [15]–[17]. This synaptic strengthening is i) specifically related to associative processes, since it is not present in subjects that received the stimuli in a temporally uncorrelated manner, ii) localized to vermal lobules V and VI, an area that receives convergence of acoustic and nociceptive stimuli [18], [19] and it is related to the expression of emotional behavior [20], iii) long lasting, since it is still present at least 24 h after learning. A similar LTP has been reported following motor learning in lobule HVI [21]. Indeed, fear memory was impaired in mutant mice with a selective dysfunction of PF-PC synapses [15]. Finally, PC-specific knockout of the protein phosphatase PP2B selectively impairs PF-PC LTP and cerebellar motor learning [22].

The basolateral amygdala (BLA) plays a crucial role in emotional memory [1], [2], [23]–[25]. It has been proposed that BLA is the site of the associative changes related to memory formation [23], [25]. Furthermore, BLA may enable learning-induced plasticity to be formed in other brain sites [1]–[2]. BLA and cerebellum may interact during memory processes [4], [26], [27]. Therefore, in the present study, we investigate the impact of BLA inactivation on cerebellar plasticity occurring during memory formation.

## Results

### Behavior

**Figure 1:**
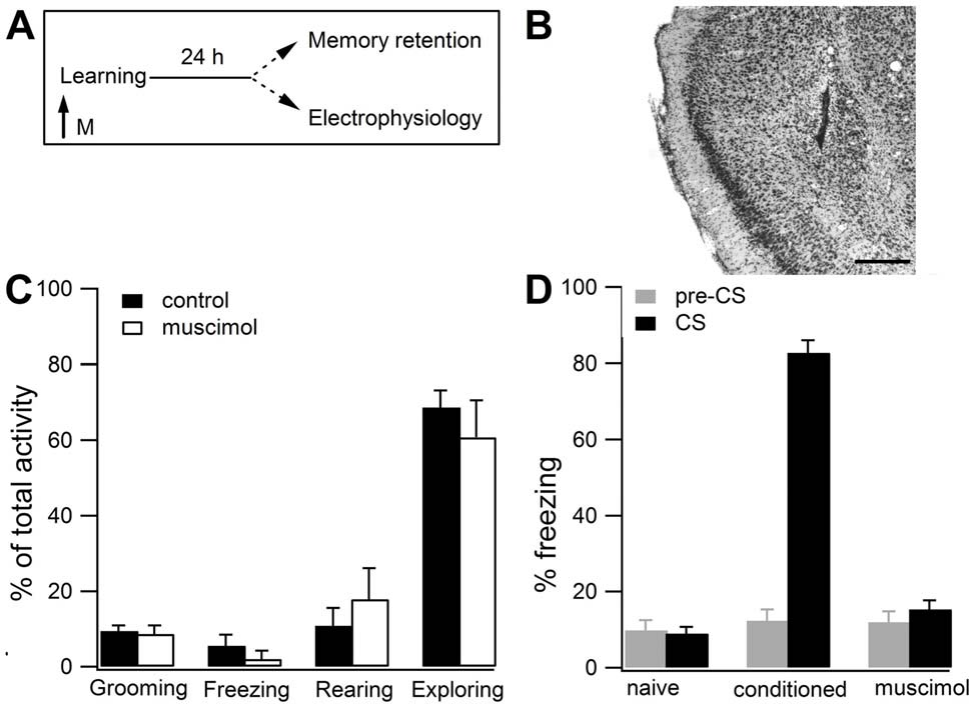
Effects of pretraining BLA inactivation on spontaneous and conditioned fear behavior. **A.** Experimental design. The arrow indicates pretraining muscimol (M) injection. **B.** Photomicrograph (magnification 4X) showing the position of the needle track in animals that received muscimol into BLA. Scale bars, 300 µm. **C.** Spontaneous activities showed by control (filled columns) and muscimol-injected (empty columns) animals before shock presentation. **D.** Long-term memory retention evaluated 24 h after conditioning by measuring freezing 2 min before (gray columns) and during CS presentation (filled columns). All values are mean ± SEM.

As a first step, we validated the experimental protocol aimed at preventing conditioned fear learning under inactivation of BLA. To block BLA without affecting the passing fibers, we used the GABAergic agonist muscimol [28]–[30] (Fig. 1A). Fig. 1B shows the position of the needle track into BLA. At the selected coordinates, the injected volume primarily inactivates BLA [31]. To inactivate BLA during fear memory acquisition, we injected muscimol one hour before training. To ensure that this procedure does not alter the spontaneous activity of the subjects, before conditioning we recorded several types of behavior that rats normally display in a new environment, namely freezing, rearing, grooming and exploring [32]. Fig. 1C shows the mean percentage activities recorded during the 2 minutes preceding the conditioning trial. Student's t-test indicates no difference between rats infused with muscimol and the control subjects for all spontaneous activities, in line with previous findings [32]. Long-term memory retention was tested one day after conditioning. At this time interval, we measured freezing response in three different groups: i) conditioned animals, which the day before received a series of pairings of tone (CS) and footshock (US), ii) naïve animals, which received no training; and iii) conditioned subjects that received muscimol before CS-US presentation. In these groups, freezing was measured during the presentation of the CS and also during the two min that precede this administration (Fig. 1D). Freezing before CS presentation did not differ among the three groups (one-way ANOVA, *F*_(2,29)_  = 0.21; NS) (Fig. 1D, gray columns), suggesting that all the employed procedures produce a very low generalized fear response [10], [24]. During CS presentation, one-way ANOVA showed a significant difference among naïve, conditioned and muscimol-injected subjects (*F_(_*_2,29)_  = 251.65; P<0.001) (Fig. 1D, filled columns). Newman-Keuls test showed significant differences between conditioned animals and those that received muscimol (P<0.05), but not between muscimol-treated subjects and the naïve ones. Thus, BLA blockade performed during CS-US presentation prevents fear memory formation, as previously reported [28], [30].

**Figure 2:**
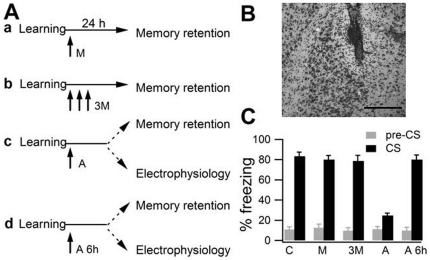
BLA reversible blockade and fear memory consolidation. **A.** BLA role in fear memory consolidation was studied by injecting into this site a) muscimol (M) 5 min after training; b) muscimol (M) 5, 90 and 180 min; c) anisomycin (A) 5 min; d) anisomycin (A) 6 h after training. **B.** Histological control of the location of anisomycin injection into BLA (magnification 10X). Scale bars, 200 µm. **C.** Memory retention tested in conditioned (C) subjects and in those that received one (M) or three (3M) injection of muscimol, anisomycin (A) 5 min or 6 h (A 6 h) after conditioning.

We evaluated the role of BLA during fear memory consolidation by injecting muscimol shortly after the acquisition (Fig. 2A). A single muscimol injection administered immediately after learning an inhibitory avoidance task prevents memory formation [33], while its administration after fear learning did not [30]. Given that muscimol effects terminates within a few hours [29], while consolidation lasts several hours and days [1], in another experimental group we prolonged muscimol activity by two additional administrations performed 90 and 180 min after acquisition (Fig. 2A).

Finally, in two additional groups, we blocked BLA protein synthesis by administering anisomycin 5 min or 6 h after conditioning (Fig. 2A). Fig. 2B indicates the position of the needle track. Fig. 2C shows freezing response in all subjects. During CS presentation, one-way ANOVA revealed significant differences among groups (*F_(_*_4,46)_  = 33.08; P<0.001). Newman-Keuls test showed differences among animals that received anisomycin shortly after the acquisition and all the other groups (P<0.05). Muscimol-treated subjects never differed from conditioned animals (P>0.05 in all cases). These data indicate that muscimol does not affect fear memory consolidation. On the other hand, the blockade of protein synthesis into BLA caused amnesia when performed 5 min, but not 6 h, after learning, as previously reported [25].

Our data are in line with previous findings showing that although pre-training functional inactivation of BLA with muscimol impaired Pavlovian fear conditioning [28], [30], immediate post-training inactivation had no effect [30]. In contrast, post-training inactivation of BLA consistently impaired inhibitory avoidance learning [33]. These results are consistent with those of previous studies in which intra-amygdala administration of AP-5 (a selective NMDA receptor antagonist) impaired Pavlovian fear conditioning if given before, but not immediately after, training [34]. In contrast, post-training infusion of AP-5 has been shown to impair inhibitory avoidance learning [35]. Collectively, the findings indicate that Pavlovian fear conditioning and inhibitory avoidance are differentially affected by post-training pharmacological manipulations of BLA and suggest that fundamental differences exist in the underlying neural mechanisms mediating memory consolidation in the two learning paradigms.

Overall, it should be pointed out that muscimol increases GABAergic activity, while anisomycin blocks the synthesis of new proteins in both glutamatergic and GABAergic neurons, i.e. these two substances have a completely different impact on the global activity of the injected site. Such a difference may be responsible of the differential effects that the two substances had on the consolidation of fear conditioned memories.

### Effects of BLA inactivation performed during fear acquisition on long-term cerebellar plasticity

**Figure 3:**
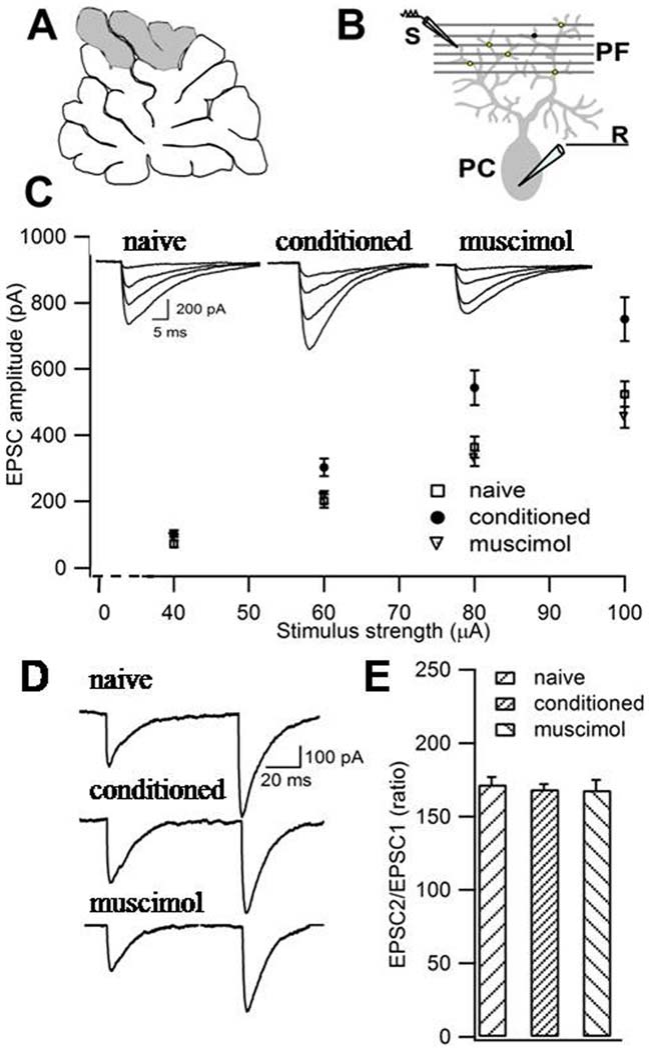
Pretraining BLA inactivation prevents learning-induced LTP in cerebellum. **A.** Electrophysiological recordings were performed on lobules V and VI (gray area) of cerebellar vermis. **B**. PF-PC EPSCs were recorded (R) at the PC soma by stimulating (S) PF in the molecular layer. **C.** Input-output data from naïve (square), conditioned (circle) and muscimol-injected (triangle) animals. **D.** Representative traces of EPSCs obtained by paired PF stimuli with 100 ms interval in naïve, conditioned and muscimol-injected subjects. **E.** Paired-pulse facilitation is similar in the three groups. All values are mean ± SEM.

We recorded cerebellar activity 24 h after muscimol injection into BLA. In vermal lobules V and VI (Fig. 3A), we analyzed the excitatory transmission at the PF-PC synapse (Fig. 3B). Fig. 3C illustrates the amplitude of the currents evoked in the PC by stimulating the PF at increasing strength. Input-output relations measuring excitatory postsynaptic currents (EPSC) amplitude (pA, output) as a function of PF stimulus intensity (µA, input) for each neuron were compared in naïve, conditioned and muscimol-treated subjects. To provide a quantitative evaluation of the response in the PC, we calculated the slope of the curves [15]. One-way ANOVA showed significant differences among the three groups (*F_(_*_2,45)_  = 18.06; P<0.001). Newman-Keuls test indicated that the averaged slope value for the conditioned group (11.60±0.9 pA/µA, *n. of cells*  = 15) was significantly higher relative to naïve (7.58±0.54 pA/µA, *n* = 16) and muscimol-treated (6.18±0.49 pA/µA, *n* = 17) groups. There was no significant difference between naïve and muscimol-treated subjects (P>0.05). Thus, BLA blockade prevents the formation of cerebellar long-term plasticity related to learned fear.

PF-PC EPSCs are characterized by paired-pulse facilitation (PPF). Such facilitation is an index of a short-term enhancement in synaptic efficacy attributed to residual calcium that facilitates transmitter release. Changes in PPF have been considered as an index of modification in PF presynaptic activity [36]. Learning-induced LTP in the cerebellum is mediated by postsynaptic mechanisms [15], [17], [36]. We employed PPF to test i) that under our experimental conditions the mechanisms that mediate learning-induced LTP have no presynaptic components and ii) that BLA inactivation has not durable effects on PF transmitter release probability. Fig. 3D illustrates the facilitation induced by pairs of PF stimulation. One-way ANOVA showed no difference among naïve, conditioned and muscimol-injected subjects (*F_(_*_2,45)_ = 0.152; NS) (Fig. 3E). The lack of difference between conditioned and naïve subjects confirms previous findings on the postsynaptic nature of learning-induced LTP [15], [17], [36]. The lack of difference between muscimol-treated animals and the naïve ones suggests that BLA inactivation has not durable effects on PF transmitter release probability.

### Protein synthesis blockade in BLA affects learning-induced LTP in the cerebellum when performed 5 min, but not 6 h, after training

**Figure 4:**
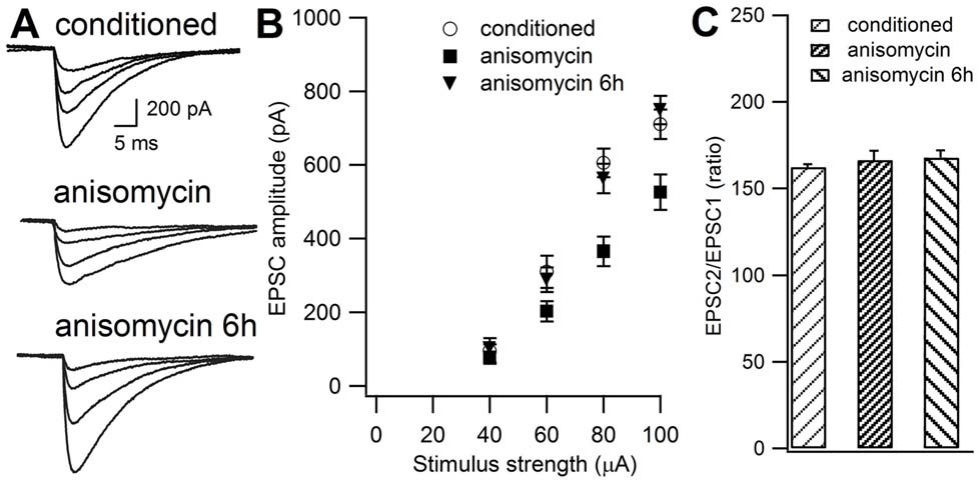
BLA inactivation during fear memory consolidation affects learning-induced LTP in cerebellum when performed 5 min, but not 6 h, after training. **A**. Representative traces of PF-PC EPSC from conditioned animals and from those that received anisomycin 5 min or 6 h after acquisition. **B**. Input-output data from conditioned subjects (circle), and those injected with anisomycin 5 min (square) and 6 h (triangle) after training. **C**. Paired-pulse facilitation in the three groups. Data are expressed as mean ± SEM.

To study the interaction between BLA and cerebellar plasticity during fear memory consolidation, we inactivated BLA 5 min or 6 h after the acquisition session by injecting anisomycin into this site. Electrophysiological recording was performed one day after BLA blockade. Fig. 4A shows PF-PC EPSC in the conditioned subjects and in those receiving anisomycin 5 min or 6 h after conditioning. Input-output relations were compared in these three groups (Fig. 4B). One-way ANOVA showed significant differences among groups (*F*(2,38) = 7.63; P<0.05). Newman-Keuls test indicated that the averaged slope of animals that received anisomycin shortly after training (7.59±0.63 pA/µA, *n* = 19) differed from the slope of conditioned group (12.64±1.35 pA/µA, *n* = 9) and of the animals injected with anisomycin 6 h later (10.61±1.01 pA/µA, *n* = 13). No difference was found between the latter two groups (P>0.05) (Fig. 4B). Therefore, BLA inactivation affects learning-induced LTP in the cerebellum when performed 5 min, but not 6 h, after conditioning. Again, we did not observe any significant difference on PPF analysis among the three groups (ANOVA test, *F*(2,38) = 0.24; NS) (Fig. 4C).

## Discussion

In the present study, we showed that during fear memorization BLA reversible blockade impairs learning-induced LTP in the cerebellum. Our findings reveal that BLA modulates cerebellar plasticity. Moreover, they suggest that the synaptic strengthening underlying learning is a heterosynaptic phenomenon that requires inputs from other neural structures.

Previous studies showed that in cerebellum PF-PC LTP is strictly related to learning processes. It is i) present in subjects that received CS and US in a temporally paired way, but not in those receiving the same two stimuli separately, ii) long-lasting, iii) localized to the lobules and synapses engaged by fear learning [15]. Mutant mice lacking PF-PC LTP were also impaired in fear memory retention [15]. In addition, the selective deletion of protein phosphatase PP2B selectively abolished postsynaptic PF-PC LTP [22]. The mutants showed impaired vestibulo-ocular reflex as well as impaired acquisition of classical delay conditioning of their eye blink response [22].

In the present work, we found that learning-induced LTP was absent in subjects that received muscimol into BLA before training. Likely, this effect is due to an interference with LTP induction occurring during CS-US presentation. However, BLA is necessary also for pain-related response [37] and for the regulation of fear innate behavior [31], [38]. Thus, although we did not observe a significant change in animals' spontaneous activity before fear acquisition, we cannot exclude an effect on CS and/or US processing produced by pretraining BLA inactivation. In line with the present findings, previous studies reported that pretraining BLA blockade attenuated activity-dependent processes in thalamus [28], [39], cingulated cortex [39], and hippocampus [32].

In a second line of experiments, we blocked BLA after learning, i.e. during the consolidation phase of memory process. This approach allows us to rule out any interference with sensory or painful stimuli processing so that any effect on cerebellar plasticity is only due to the interference with the memory trace. To date, the only study that tested the effect of BLA inactivation on activity-dependent processes that occur in regions engaged in consolidating long-term memories has been performed by McIntyre et al. (2007). The authors showed that post-training infusion of lidocaine into BLA significantly reduced the increase in Arc protein observed in hippocampus following avoidance learning [40]. Our study extends these results to the long-term synaptic plasticity, i.e. LTP, which underlies memory formation in the cerebellar cortex.

In the hippocampus and cerebellum the electrically-induced LTP is widely considered a cellular model of learning. A support to this hypothesis comes from recent findings showing that learning-induced LTP interferes with the subsequent electrically-induced LTP in hippocampus [41]–[43] and cerebellum [17]. The present results, however, reveal an important difference between electrically- and learning-induced LTP. Namely, the latter type of LTP requires information from other regions to be formed and maintained. Hebbian model of learning maintains that pre- and postsynaptic neurons have to be coactive within a defined time period to modify synaptic strength. In our model, CS and US reaching the cerebellar cortex produce LTP provided that a heterosynaptic input coming from BLA sets the proper local conditions of such an interaction. Thus, studies employing the electrically-induced LTP in order to identify the cellular mechanisms related to memory processes should take into account the heterosynaptic inputs, considering them as integrative units. Theoretically, the functional meaning of the heterosynaptic-dependence of learning-induced LTP might be that local synaptic processes underlie the automatic recording of an attended experience. During this time period, structures elsewhere evaluate the emotional content of such experience, and, as appropriate, transform it via heterosynaptic stimulation into long-term memory traces.

In line with the present data, in hippocampus a weak tetanic stimulation, which ordinarily leads to an early potentiation lasting less than 3 hours, results in an LTP lasting for at least 8 hours, when a repeated tetanization has already been applied at another heterosynaptic input to the same population of neurons [44]. In vivo, such an early potentiation is transformed into a LTP by appetitive and aversive stimuli [45] and this reinforcement is blocked in BLA-lesioned animals [46]. Accordingly, BLA stimulation facilitates the electrical induction of LTP in hippocampus [46]–[48], thalamocortical system [49], and striatum [50], while the inactivation of BLA decreases LTP in hippocampus when performed during, but not 20 min after, the application of the tetanus [48]. On this ground, our results provide the first evidence that the synaptic strengthening occurring during memory trace formation is heterosynaptic in nature and requires BLA activity.

To date, there is no evidence of a direct anatomic pathway connecting BLA to the cerebellum. Thus, BLA may influence cerebellar plasticity via two mechanisms: first, this site may regulate cerebellar level of monoamine, like noradrenalin, serotonin and dopamine. It is known that BLA modulates the influences of adrenal stress hormones on memory consolidation [1], [2]. Indeed, monoamine signals are involved in cerebellar learning [51]. The other possibility relies on the fact that BLA sends direct projections to brain sites that in turn act on cerebellum. For instance, during eye blink conditioning, it has been proposed that BLA exerts an excitatory influence on cerebellum via the lateral tegmental field [26]. In addition, BLA is anatomically connected with the hypothalamus, a region that is bidirectionally connected with the cerebellar vermis [52].

Several findings support the involvement of the cerebellum in learned fear. In humans, the cerebellum is strongly activated during mental recall of personal fear-related events [11] and by associating sensory stimuli with a painful stimulation [13], [14]. Changes in heart rate induced by repeated pairing of CS and US are hampered in patients with medial cerebellar lesion [9] and in animals with vermal lesions [7], [8]. In all studies, these effects are due to an interference with associative processes, because baseline responses to CS and US are not affected. In addition, the reversible inactivation of cerebellar cortex during memory consolidation impairs fear memory retention [10]. This result has been obtained by blocking this site after training and by performing the retention trial when the reversible blockade was over, i.e. with no interference with sensory or motor response. However, the role played by the vermis in fear conditioning remains to be clearly defined. Given its well known role in associative motor learning [53], [54], it has been suggested that the cerebellum coordinates the adequate motor response [4],[26]. However, by way of the fastigial nucleus, vermis is connected also to the hypothalamus, to periaqueductal gray area, the locus coeruleus and the ventral tegmental area, thus it can regulate the cardiovascular tone, respiration, gastrointestinal functions, as well as other autonomic processes [55], [56]. In addition, the vermis is also connected with brain sites that are associated with affective and learning processes, like BLA and hippocampus [5], [6], [55]. Indeed, vermian cortex and fastigial stimulation induces electrophysiological responses in BLA, in septum and hippocampus in cat [57], rats [58] and monkeys [59] and fear-related responses are elicited during electrical stimulation of the vermis [55], [57]. Therefore, it may be that cerebellum is involved in fear learning in order to set the more appropriate responses to new stimuli and/or situations [11], i.e. this site may translate an emotional state elaborated elsewhere into autonomic and motor responses [6]. In this context, learning-induced LTP at PF-PC synapses may enable the CS to activate PC and thus to trigger the more adequate autonomic and behavioral responses to the CS. Further studies, however, should better verify this hypothesis.

## Materials and Methods

### Subjects

We employed P30-P33 male Wistar rats (Harlan, Italy). The animals were housed in plastic cages with food and water available ad libitum, under a 12 h light/dark cycle at a constant temperature of 22±1°C. All animals care and experimental manipulations were conducted in accordance with the European Community Council Directive of November 24, 1986 (86/609/EEC) and approved by the Bioethical Committee of Turin University.

### Surgery and drugs administration

Bilateral guide cannulae were implanted dorsal to the BLA one week before the behavioral and electrophysiological procedures. Rats were anesthetized with ketamine (100 mg/kg; Ketavet; Bayer, Germany) supplemented with xylazine (5 mg/kg; Rompun; Bayer, Germany) and mounted in the stereotaxic apparatus. Bilateral cannulae were implanted to a depth of 2 mm from skull at the following stereotaxic coordinates: anteroposterior, -2.1; mediolateral, ±4.0 mm from bregma, as in a previous work [31]. The cannulae (outside and inside diameters, 0.6 mm and 0.4 mm, respectively) were secured to the skull with dental cement and closed with mandrels smeared with mineral oil. Before injection, the animals were restrained by hand, the mandrel was removed and replaced with an injection needle (outside diameter, 0.3 mm) connected with a short piece of polyethylene tubing to a Hamilton syringe. The needle was equipped with a stopper that limited the depth of insertion to 7.0 mm beyond the tip of the guiding cannula. After the solutions (muscimol or anisomycin) had been injected over a 1 min period, the needle was left in place for an additional 1 min before being slowly withdrawn. Control subjects were designed to mimic the infusion procedure without causing any possible disturbance to BLA. Since BLA activity and fear behavior are affected by saline injection into BLA [38], [60], in control subjects the needle was lowered 2 mm above the BLA, without infusing fluid, for 2 min.

To block BLA during fear learning, one hour before training, we injected 0.3 µl of a 2 mg/ml GABA agonist, muscimol, (Sigma-Aldrich) into BLA. To interfere with fear memory consolidation, we injected into BLA i) muscimol (0.3 µl, 2 mg/ml) shortly after training; ii) muscimol three times following the acquisition session, i.e. 5 min +90 min +180 min afterwards; iii) 0.3 µl of a 62.5 µg/0.5 µl of the protein synthesis inhibitor, anisomycin (Sigma-Aldrich) shortly after training or iv) 6 hr later.

### Behavioral procedures

Conditioned fear responses were obtained as in our previous studies (40,60). Briefly, the subjects were placed in a basic Skinner box module (Coulbourn Instruments, Allentown, USA) and left undisturbed for 2 min. Then, a training session consisting of 7 presentations of tone (7 s, 1000 Hz, 70 dB) (CS), coterminating with an electric foot shock (2 s, 1 mA) (US), was delivered with intervals of 30 s. immediately afterwards, the animals were returned to their home cage. Cued fear retention was evaluated 24 h after conditioning in a totally new context, in order to avoid the facilitation of CS retention caused by the contextual cues [24], [41]. The box was located in a different room from that of the initial training. After 2 min of free exploration, a series of 7 acoustic stimuli (CS) were administered, identical to those used during the acquisition. Rat's behavior during conditioning and retention testing was recorded by means of a videocamera. Freezing response, defined as the complete absence of somatic motility except for respiratory movements, was taken as a fear index. Measurements were performed by means of a stop-watch by personnel that did not known to which experimental group each animal belonged. Total cumulative freezing time (i.e. total seconds spent freezing during each chosen period) was measured and calculated as a percentage of total time. All behavioral procedures were performed between 9.00 a.m. and 12.00 a.m. to minimize circadian influence.

### Electrophysiological recordings

Parasagittal cerebellar slices (200 µm thick) were prepared 24 h after the acquisition trial following standard procedures [15], [17]. Whole-cell patch-clamp recording was performed from Purkinje cell (PC) soma on vermal lobules V-VI. PCs were held in voltage-clamp mode at a holding potential of −70 mV. Bicuculline (20 µM) (Tocris Cookson, UK) was applied in the perfusate to inhibit GABAergic activity. Patch pipettes (3–4 MΩ) pulled from borosilicate capillary were filled with an intracellular solution containing (in mM): 120 CsCl, 20 TEA, 10 HEPES, 4 Na_2_ATP, 0.4 Na_3_GTP, 2 MgCl_2_, 10 EGTA, pH 7.3 adjusted with CsOH. An 80 ms, −4 mV test hyperpolarizing pulse preceding each stimulus was delivered to monitor the series and input resistances of the PC throughout experiments. Series resistance and input resistance were evaluated by measuring the negative peak amplitude and the steady-state amplitude respectively from the response to the preceding pulse. Recordings were discarded from the analysis if the leak current exceeded −500 pA, or if the input resistance changed significantly or if the series resistance changed by more than 20%. PFs were stimulated with 100 µs pulses delivered by an isolated pulse stimulator (A-M Systems, USA) through a glass pipette filled with ACSF placed in the external half of the molecular layer. Excitatory postsynaptic currents (EPSCs) evoked in the PCs by PF stimulation were recorded. Negative current pulses ranging from 40 to 100 µA with duration of 100 µs were delivered in ascending and descending order at 20 s intervals. EPSC amplitude was measured as the difference between the current baseline level before the stimulus artifact and the peak of the EPSC. For each stimulus intensity, a single EPSC value was calculated as the mean of six EPSCs evoked by ascending and descending stimulus intensities. The values of every cell, recorded within each group of rats, were used to calculate means, S.E.M. and statistical tests. One-way ANOVA-test was performed on the slope values of the linear fits obtained in each cell for the first three points of the stimulus-response curve. To establish the pre- or postsynaptic origin of changes in synaptic strength, pairs of pulses of 60 µA separated by 100 ms were delivered. The paired pulse ratio of the second to the first EPSC amplitude was then calculated. Typically, 2–3 cells were recorded per animal, with one or two neurons per slice. Data were acquired using Pulse software (HEKA Elektronik, Germany) and analyzed offline with the program Igor Pro (Wavemetrics, USA).

### Histology

Injection needle tracks were identified in Nissl-stained serial sections following standard procedures [41]. Only those animals showing a correct needle placement in the target site were included in the analyses.

### Data analysis

Student's t-test, one-way ANOVA and Newman-Keuls test were used.
